# Criminal Jurisprudence Considered in Relation to Mental Organization

**Published:** 1842-07-01

**Authors:** 


CRIMINAL JURISPRUDENCE CONSIDERED IN RELATION TO MENTAL ORGANIZATION. By *M. B. Sampson*. Octavo, in double columns, pp. 30. Highley, Fleet Street. 1842.

THESE letters, six in number, were originally published in the Spectator; but are now collected into a pamphlet, and sold at the cheap price of sixpence, not with the view to profit, but to the extensive dissemination of the principles which the author maintains. This code of criminal jurisprudence is founded mainly on phrenology, and on that account will create a prejudice in the minds of many well-meaning persons, as tending to Materialism, and contrary to the doctrines of Christianity. But as the laws under which we live are framed entirely for the punishment of crimes in this world, and have no reference whatever to rewards or punishments in a future state of existence, people of the most tender or scrupulous conscience may examine the principles in these letters as bearing on criminal jurisprudence, and cull from them such hints as may appear to be founded on just reasoning, or drawn from authentic facts.

The author sets out by taking it for granted, and as an unquestionable truth, that "all the *manifestations* of the mind, including the feelings and the passions, are dependent on the formation and state of health of its material instrument the brain." This must, we think, be granted, since it is impossible that the soul can manifest itself, except through the instrumentality of matter. The next proposition is, that diseases or disorders of the cerebral *structure* will give rise to functional disorders of the mind's organ, and consequently of the mental manifestations. We fear that every close observer will see too much truth in this second proposition; for not only cerebral, but hepatic, gastric, and intestinal disorders or irritations will disturb or even completely destroy, for a time, the *healthy* manifestations of the mind.

"The question then arises, why do we not treat irregularities of the mind in the same way as we treat all other physical disorders, viz. by confining ourselves solely to an attempt to cure the patient; and why do we talk of punishment when we are considering a case of morbid action of the brain, any more than when we are considering a case of morbid action of the heart, the lungs, or any other organ?" 5.

The great difficulty is to define the limit between sanity, with responsibility, and insanity, with irresponsibility. What renders the difficulty greater is the fact that, there is scarcely a human being in this world in whom "*all* the qualities of mind and body are healthfully constituted and harmoniously developed." This tallies with religion and daily observation; but some come much nearer the standard of perfection than others.

"The tendency to evil, which, more or less, is the characteristic of all men, indicates in each the amount of this divergence from that harmonious balance of the mental powers in which alone true soundness of mind can consist. False impressions, ungovernable desires, deficiencies of intellect or feeling—in short, all that makes up the sum of human errors—arises from an unbalanced action of the various faculties of the mind; and to the extent, therefore, that any one faculty is deficient in its comparative relation to the others in any individual, such is the extent of his departure from true soundness of mind in regard to those objects to which that faculty may relate." 6.

Thus if the tendency to acquire property is not greater than the tendency towards benevolence, conscientiousness, &c., the property will be fairly acquired in accordance with justice and humanity; but if the tendency in question be inordinate, he will endeavour to acquire property by all means, whether just or unjust. Now as the perfect type is no where to be found in mankind, how are we to estimate the *amount* of departure from that type? The author proposes a rule for that purpose. He considers obedience to the laws of his country as a test of sanity.

"Although, therefore, it may be asserted that, in the eye of perfection, there exists no human mind in its right state, yet so long as an individual infringes no general law or habit of society, he may be considered as coming up to the average point of civilization, and may therefore be regarded as perfectly sane." 6.

It is probable that we shall not be able to find a more practical rule than the above. But still we fear there must be admitted a kind of neutral ground between this standard and unequivocal insanity—on which stand the innumerable *eccentricities*, both of opinion and action, which break no law or ordinance of society, yet appear as obliquities of intellect. This neutral ground is denied by the author.

"Irregularities of disposition arise from two causes,—viz. the transmission of an irregular cerebral organization from parent to child; and, subsequently, the effect of accidental circumstances, as bad example, ill-conducted education, injuries of the head, &c. It is precisely from analogous causes that irregular conditions are occasioned in other organs of the body. They are more or less, in all cases, transmitted in an imperfect and unhealthy state; and the subsequent effect of defective physical education and accidents aggravates the predisposition to morbid action which was thus originally established. If a child is born with an irregular organization of brain (and to say that every child is born thus, is merely to aver that none are born perfect,) he comes into the world to the extent of that irregularity insane; and as, by subsequent education, that irregularity may be reduced or increased, so will this insanity be aggravated or relieved." 6.

We are much more inclined to agree with Dr. A. Combe in the following sentiment.

"Many men in the full enjoyment of health are remarkable for peculiarities of character and idiosyncracies of thought and feeling which contrast strongly with the general tone and usages of society; but they are not on that account to be held as insane, because the singularity for which they are distinguished is with them a *natural* quality. *It is the prolonged departure, without an adequate external cause, from the state of feeling and modes of thinking usual to the individual when in health, that is the true feature of disorder of mind.*" 7.

No doubt this definition is not free from objections and difficulties; but no definition of sanity or insanity will ever be free from them.

It will be a long time before the world comes to the conclusion that "the frame of mind which leads to criminal acts should invariably be attributed to a *derangement* of the mental *organs*, or to a defect in their structure."

Admitting that this was physiologically and pscychologically true,—admitting that a person can no more resist the impulse to crime than he can the impetus of a bullet from a cannon's mouth—what are we to do with a Daniel Good—what ought we to have done with Greenacre and Courvoisier? Allowing them to have been insane, and that they could not resist the evil impulse, they ought not to have been punished. But confinement itself is punishment, and to that we must resort, in order to protect society from murders and robberies. We do not advocate the punishment of *death* for any crime—even for murder—*but* the punishment which effectually secures the public, and gives the culprit (if that term may be applied) the best chance of being reclaimed—namely, confinement or hard labour, with privation of all comforts—and, *à fortiori*, of all pleasures. The following passage is ingenious "special pleading" in the physiological court.

"When a man commits a crime, it is the custom to exclaim that 'he *ought* to have known better.' Now, if he was, from natural deficiency of the reasoning or moral powers, unable to perceive that he was doing wrong, it cannot be disputed that he was of unsound or partially idiotic mind. If, on the other hand, he did possess the power to perceive the right course, and yet was unable to act up to his conviction, it is evident that he possessed a brain of such an irregular formation, that the higher mental powers bore no sufficient relation to the lower propensities which it is their duty to control; and that the latter, when roused by the presentment of their own stimuli, possessed a strength so disproportionate as completely to overpower the former. If, while in this state, he commits a crime, he will exclaim that 'he could not help it,' or that the Devil (*i. e.* the cerebral organ of the offending propensity) was too strong for him. His judgment, in fact, was strong enough, under ordinary circumstances, to teach him the erroneous tendency of his passions: but it was not strong enough to prevent his falling, when those passions, always disposed to disproportionate action, became suddenly excited by some external cause. In such cases, the mental balance is completely lost, and he is reduced to a state of relative insanity. Under these circumstances, the 'responsibility' which attaches to the result of his conduct should be (and under the operation of the Divine laws certainly is) shared by those who, being too ignorant to estimate the nature of his infirmity, suffered the exciting causes to be placed in his way, instead of endeavouring to repress the activity of the overruling propensity by withholding the objects of temptation, and by appealing to his higher but hitherto neglected powers." 8.

Mr. Sampson goes on to aver that there is no merit in good actions, nor culpability in bad ones—both being the result of organization, which was not in the choice or power of either party. The author quotes a passage from the writings of the celebrated Jeremy Taylor, the great ornament of the church, which certainly tends much to uphold his argument, however revolting it may appear to our feelings.

"If a man be exalted by reason of any excellence in his soul, he may please to remember that *all souls are equal;* and their differing operations are because their *instrument* is in better tune, and their body is more healthful or better tempered; which is no more praise to him than it is that he was born in Italy. On the other hand, if his course entitles him to no reward in this world beyond the natural one of the inevitable happiness of mind which Heaven has decreed to be the consequence of its *physical health*, so it is but fair to allow that the opposite course can merit no punishment beyond the inevitable pain which Heaven has decreed to be the consequence of its *physical* derangement." 6.

It must be obvious to every impartial reader that a more completely phrenological doctrine than the above is not to be found in the writings of Gall, Spurzheim, Combe, or Sampson. But we apprehend that His Reverence went rather farther than he intended in the foregoing passage. If virtue and vice—if good and bad actions—have neither rewards nor punishments in this world, *because* they flow inevitably and irresistably from organization, how can we believe that the omniscient and benevolent Author of our existence should punish or reward us for "deeds done in the flesh," seeing that we had no more control over those deeds than we had over the motions and revolutions of the heavenly bodies in the firmament? And again;—if there be no rewards or punishments in a future state, of what use or to what purpose can a future state be? It can have no relation to a *preceding* state of existence, and therefore, if it take place at all, it must, to all intents and purposes, be a new creation, and totally unconnected with this, our mundane being! These are serious, but inevitable conclusions from the tenets of Jeremy Taylor.

An æra, indeed, may come, when our descendants may be prepared for such doctrines, and when they will look back with pity and contempt on the superstitious fears and hopes with which the minds of their forefathers were filled respecting an immortality—a heaven and a hell—a paradise and a purgatory—that existed only in the reveries of imagination! This æra, however, is not yet come; and, if we can trust to appearances, it is a long way off. We cannot, therefore, congratulate Jeremy Taylor, or our present author on any prospects of success in the propagation of their doctrines—at least in the present century.

In our author's second letter, he brings forward several cases of suicidal and homicidal monomania—in which the destructive propensity was so strong that the individuals were quite unable to resist, and, in many instances, desired to be secured, lest they committed the act, which they knew to be criminal, though their reason was unable to quell the impulse. Such cases are familiar to all medical practitioners, and form strong arguments in support of Phrenology, or the plurality of organs in the brain.

Our author observes, that one organ may grow larger than it ought naturally to be, by an over-supply of nutriment—a kind of hypertrophy, with a "permanent increase of sanguiferous circulation in the region of the organ." This again may lead to structural change in the part, and incurable insanity. In recent cases, there is seldom change of structure, but only increase of vascularity in the part, especially under excitement.

"If a person is under the slightest excitement, nay, under the mere operation of any ordinary feeling, an increased supply of blood for its manifestation is required and sent to its organ in the brain. If the emotion increases, the supply of blood to its organ must have increased in a due proportion, and so on, until the supply becomes so great as to carry it to a state of blind excitement that may cause it to overpower all other emotions, and eventually even lead it to act without the knowledge of the intellect, or the concurrence of the other feelings." 9.

Some curious examples are given, where very trifling irritations, such as a person treading on the toes of a man in this condition, have led to the most fatal actions.

The following passage is very sensible.

"He, who neglects the laws of health by exposing himself, say to a sudden and violent change of atmosphere, and has thereby produced a pulmonary affection, has to submit to the restraint of confinement at home, or to a temporary exile in a warmer climate, to remedy the evil effects of his disobedience; or if, by incautiously venturing into an impure air, he has contracted an infectious fever, and he should, nevertheless, refuse to take measures for his recovery, it would be the duty of society, both to themselves and to him, forcibly to remove him to a better atmosphere, to keep him secluded from all to whom there might be danger of his communicating the disease, and to enforce the administration of proper remedies. In like manner, if he offends against the moral laws from hereditary disposition, and the contagion of bad example, or from any other cause, it becomes the duty of society to remove him from the source of contagion, and from the means of transferring it to others; to repress the unhealthy tendency of the mind, and to stimulate its deficient organs.

But although religion, justice, and benevolence, point to this as the chief, nay, the only duty which should be regarded by society in the treatment of offenders, it is one which, in the blind and popular eagerness for the infliction of 'punishment,' is almost invariably lost sight of; and, as a natural and inevitable consequence of this neglect, details of the most disastrous kind are day by day forced upon our attention." 13.

The great difficulty is to ascertain the point at which mental aberration may render restraint necessary. Thus a man evincing an inordinate love of gambling, by which his family may be ruined, and himself brought to a state of suicidal determination, is in as much need of restraint as he who evinces a disposition to murder by sticking pins through flies, or hanging mice, for the pleasure of witnessing their dying agonies! But the present temper of society would not bear that a man should be put into a lunatic asylum for gambling. Where would the doctrine end? There are a thousand ways of gambling. Mining speculations, joint-stock companies, &c. &c. &c. by which thousands are daily ruined, might be brought under the law and doctrine in question. The following position is reasonable.

"In dangerous cases, where a mitigation cannot be effected to the requisite extent, so long, indeed, as there exists cause of apprehension of bad results from the disordered person holding communication with others—it must always be necessary to keep him in a state of seclusion apart from temptation. This will obviate the objection that my views would leave all men to follow their inclinations. Punishment from man is not necessary; when a patient is suffering from fever, we do not attempt to 'punish' him, but we keep him in seclusion from all but his medical attendants (who run little risk of infection), and we oppose his irrational desires, control his actions, and if necessary, perform painful operations." 15.

The fourth letter goes more into practical detail than general speculation or theory.

At the Eastern State Penitentiary of Pennsylvania, the following plan, which we greatly abridge, has been adopted. The convict, on his entrance, is washed, cloathed, and conducted blindfold to his cell, where he remains locked up, and abandoned to that solitary anguish and remorse which follows the commission of crime.

When labour is employed, it is not as a punishment, but as an alleviation of his sentence. The want of occupation in solitude produces a feeling of tedium and irksomeness, that is relieved by labour, which then becomes an enjoyment—which, by association, continues after the convict has left his asylum.

Persons duly qualified are employed to teach the convicts trades and avocations, as well as religion and morality. The prohibition of intercourse with society is not entirely enforced—but only amongst the convicts themselves, so that they can neither impart nor imbibe contamination. It is said that this system has worked better, and produced more salutary results than any other. The following passage is worthy of extraction.

"It will be seen, from what I have stated, that so far from being the advocate of a sentimental humanity, which turns with horror from the contemplation of that law of the Creator by which pain is rendered consequent upon misconduct, I advocate a *severer* system than that which at present obtains, since I assert that the most severe pain which can be inflicted upon any offender, is precisely that pain which results from a philosophical treatment for his cure. It is a treatment which the patient would ever afterwards remember with mingled feelings of gratitude and terror,—gratitude for the improvement which it has wrought upon his nature, and terror at the remembrance of the prolonged and bitter struggle by which that improvement was attended. The difference between the system which I advocate, and that which is at present in force (if the vague and contradictory treatment of offenders, which is now practised, can be called a system), is simply this, that I advocate a discipline which should benevolently produce great pain at first, with the view of preventing much greater pain, which must otherwise inevitably be endured for the future; while at present we revengefully inflict pain in a lesser degree, which is productive of little future benefit to the sufferer—leaving, indeed, his disorder generally unmitigated, and oftentimes increased." 18.

In respect to capital punishment, the opinion is daily increasing that it is unnatural, and inefficient, even for murder. What is it but legal murder after all? A man takes away the life of another. Let the example be followed, and let the murderer be hanged. This, no doubt, is the "lex talionis," but it is neither merciful to the culprit nor protective to society. It leaves no *locus penitentiæ* to the criminal—it renders callous the feelings of the multitudes who flock to the scene of execution—and brutalizes them by familiarizing them with the annihilation of human life. People may quote Scripture about life for life—an eye for an eye—and a tooth for a tooth—but let them adhere to the whole code of "LEX TALIONIS," or abandon it entirely. If the great object of punishment for crime be the prevention of its repetition, hanging or decapitation is the worst of all preventives. Allowing, for argument's sake, that a hanging scene produced no other effect than a horror of the crime, and a terror of the punishment; still those impressions are fugitive, and entirely forgotten in a few days. But suppose a Burke, a Greenacre, a Courvoisier, and other culprits were still labouring on the highways, with chains on their limbs, brands on their shoulders, and solitude and ascetic diet in their vacations from toil; these perpetual mementos of turpitude and its consequences would prove infinitely more preventive of crime than the momentary agonies of a wretch suspended by a rope at the New Drop. Degrading as the act of flogging is to a soldier or sailor, the disgrace of it deters far more from insubordination than the bullet from the mouth of a French musket. There is a kind of honour attached to death from a dozen of balls, whilst the ignominy of 500 lashes, which spares life, leaves a standing warning against the committal of a similar crime.

"The punishment of man consists in the infliction upon him of a treatment which is in opposition to his desires. Pleasure arises from the gratification of his desires; pain is the result when they are offended. If a man desires above all things to gratify the tendency to destroy, which results from the activity of a faculty common to his race, it being at the time in a state of excitement so great as to overmaster the dictates of all his other and higher powers, and to act independently of them, the idea that, in gratifying it, he incurs the risk of self-*destruction*, is that which of all others would be least distasteful to him. That, under such circumstances he might even contemplate it with pleasure, is shown by the large proportion of cases of murder which are terminated by the suicide of the criminal. The tendency to destroy is one of the blind propensities of man's nature, absolutely necessary to adapt him to his relation to the external world; and, when acting harmoniously with the intellect and moral sentiments, it produces only the most beneficial results; but, when roused to unbalanced action, it exhibits itself in maniacal fury, and, overpowering the reason and the feelings (which it must do before its possessor can commit murder), derives oftentimes as much pleasure from the destruction of its possessor, as from the destruction of any other individual. It gives in its morbid state an inordinate tendency to violent *action*—a wild desire to overpower restraint of every kind, and to break down and destroy all that comes within its reach. To one, therefore, who is labouring under this feeling, the present sanguinary law acts rather as a stimulant. The only thing that would at all operate with preventive force upon a mind in this state, would be the impression, that, if the organ should be gratified up to the point of homicide, it would subject its possessor to a life of perpetual RESTRAINT." 18.

That execution scenes tend to harden the mind rather than horrify the feelings, may be inferred from a fact stated by Ewart in the House of Commons, namely that, out of 167 persons who had been hanged, in a certain period of time, 164 had been present at executions previously!! The ordinaries of Newgate affirm that is very rare for any one to suffer at the Old Bailey, who had not been a witness of the sufferings of others at a similar place. When Bishop and Williams were executed, in 1831, the multitude rent the air with their groans and hisses, when the culprits were led forth on the scaffold; but the moment the drop fell, they gave *several tremendous cheers!* So much for the sight of capital punishments! It cannot be said of hanging or decapitation, that—

"Emollit mores nec sinit esse feros."

One of the most specious objections to the abolition of capital punishment is the fear that murder might often be added to other crimes—as robbery for instance. But this argument is untenable. The crime of murder should invariably be imprisonment for life, without any exception, whereas other crimes might be open to terminable restraint, according to the nature of the case or the recovery of the culprit from his vicious tendency. This plan would effectually check the wanton addition of murder to robbery or theft.

"In conclusion, I may be permitted to repeat, that the true object of all criminal laws should be simply to remove offenders from the power of gratifying the special tendencies from the action of which their errors of conduct may have arisen, and at the same time to stimulate those faculties which have hitherto lain dormant and inefficient. This must in all cases be the most painful operation that the criminal could undergo; but the object should be, by enlightening the minds of those who are doomed to suffer it, to show that it is undertaken with no feeling of vengeance, but with the same certainty of producing a good result to the patients themselves, as would be felt in medically administering a specific for any ordinary disease. They should be taught to feel that the *cure* of the depraved mind (or, to speak more correctly, of its disordered instrument), is the only thing that is aimed at, and that an eventual increase of comfort to themselves must be the result of the pain which is inflicted; that the desire is not to administer punishment, but the reverse—to see, in fact, how far they can be saved from punishment by an effort to produce the cure or mitigation which is benevolently desired, by the infliction of the least possible amount of pain. It is happily known, that when those who are suffering from any unfortunate tendency of mind can be made to see and understand an intention of this sort, many an offender will voluntarily submit to the necessary discipline." 26.

We recommend this cheap pamphlet, containing the matter of a middle-sized octavo volume, and only sixpence in price, to our readers, with an assurance that they will find in it much food for reflection.

